# Characterization of the Intramolecular Interactions and Regulatory Mechanisms of the Scaffold Protein Tks4

**DOI:** 10.3390/ijms22158103

**Published:** 2021-07-28

**Authors:** Balázs Merő, Kitti Koprivanacz, Anna Cserkaszky, László Radnai, Virag Vas, Gyöngyi Kudlik, Gergő Gógl, Péter Sok, Ádám L. Póti, Bálint Szeder, László Nyitray, Attila Reményi, Miklós Geiszt, László Buday

**Affiliations:** 1 Research Centre for Natural Sciences, Institute of Enzymology, 1117 Budapest, Hungary; senpaicorp@gmail.com (B.M.); kitti.koprivanacz@gmail.com (K.K.); laszlo.buday@eok.sote.hu (A.C.); lradnai@gmail.com (L.R.); vas.virag@ttk.hu (V.V.); Buday.laszlo@ttk.mta.hu (G.K.); szederbalint@gmail.com (B.S.); 2 Department of Biochemistry, Eötvös Loránd University, 1117 Budapest, Hungary; goglg@igbmc.fr (G.G.); nyitray@elte.hu (L.N.); 3 Research Centre for Natural Sciences, Institute of Organic Chemistry, 1117 Budapest, Hungary; sok.peter@ttk.hu (P.S.); poti.adam.levente@ttk.hu (Á.L.P.); remenyi.attila@ttk.hu (A.R.); 4 Department of Physiology, Semmelweis University, 1094 Budapest, Hungary; geiszt@eok.sote.hu; 5 Department of Molecular Biology, Semmelweis University Medical School, 1094 Budapest, Hungary

**Keywords:** Tks4, Tks5, p47, SH3 domain, tandem SH3, PX domain, autoinhibited conformation, SAXS, MST, ITC, lipid binding, PtdIns(3)P, scaffold proteins

## Abstract

The scaffold protein Tks4 is a member of the p47^phox^-related organizer superfamily. It plays a key role in cell motility by being essential for the formation of podosomes and invadopodia. In addition, Tks4 is involved in the epidermal growth factor (EGF) signaling pathway, in which EGF induces the translocation of Tks4 from the cytoplasm to the plasma membrane. The evolutionarily-related protein p47^phox^ and Tks4 share many similarities in their *N*-terminal region: a phosphoinositide-binding PX domain is followed by two SH3 domains (so called “tandem SH3”) and a proline-rich region (PRR). In p47^phox^, the PRR is followed by a relatively short, disordered *C*-terminal tail region containing multiple phosphorylation sites. These play a key role in the regulation of the protein. In Tks4, the PRR is followed by a third and a fourth SH3 domain connected by a long (~420 residues) unstructured region. In p47^phox^, the tandem SH3 domain binds the PRR while the first SH3 domain interacts with the PX domain, thereby preventing its binding to the membrane. Based on the conserved structural features of p47^phox^ and Tks4 and the fact that an intramolecular interaction between the third SH3 and the PX domains of Tks4 has already been reported, we hypothesized that Tks4 is similarly regulated by autoinhibition. In this study, we showed, via fluorescence-based titrations, MST, ITC, and SAXS measurements, that the tandem SH3 domain of Tks4 binds the PRR and that the PX domain interacts with the third SH3 domain. We also investigated a phosphomimicking Thr-to-Glu point mutation in the PRR as a possible regulator of intramolecular interactions. Phosphatidylinositol-3-phosphate (PtdIns(3)P) was identified as the main binding partner of the PX domain via lipid-binding assays. In truncated Tks4 fragments, the presence of the tandem SH3, together with the PRR, reduced PtdIns(3)P binding, while the presence of the third SH3 domain led to complete inhibition.

## 1. Introduction

Scaffold proteins have pivotal roles in cellular signaling by providing an assembly platform for various proteins and by specifying the subcellular localizations of the resulting protein complexes [1,2]. They usually comprise multiple domains and docking sites that can recruit appropriate sets of proteins [3,4]. One typical structural element of scaffold proteins is the SH3 (Src homolog 3) domain. SH3 domains bind short proline-rich sequences with the general consensus motif “PXXP”, where X denotes any amino acid residue [5]. SH3 domains mediate protein–protein interactions [6] or intramolecular interactions within the same protein [7]. Other domains, such as the PX (Phox Homolgy) domain, anchor scaffold proteins to the cell membrane by binding to phosphorylated inositol lipids [8,9].

In several scaffold proteins, the *N*-terminal PX domain is often followed by one or more SH3 domains [8,9]. This typical domain organization can be observed in all five members of the p47^phox^-related organizer superfamily [10,11]. In p40^phox^, p47^phox^, and NOXO1, the PX domain is followed by one or two SH3 domains. These proteins regulate the assembly and activation of NADPH oxidase complexes [12]. Two recently discovered members, Tks4 (also known as Fad49, HOFI, or SH3PXD2B) and Tks5 (FISH or SH3PXD2A) are named based on the early observation that they serve as tyrosine kinase substrates of Src kinase and contain four or five SH3 domains, respectively [13,14]. The PX and SH3 domains are connected by unstructured regions in all members of the p47^phox^-related organizer superfamily.

Both Tks4 and Tks5 have roles in cell motility by affecting the formation of podosomes and invadopodia [13,15,16]. However, despite the structural similarities, their functions are not totally overlapping [15,17]. In Src-transformed fibroblasts, only Tks4 recruits membrane type-1 matrix metalloproteinase to podosomes, thus playing a role in extracellular matrix degradation in these migrating cells [13]. In melanoma cells, both Tks4 and Tks5 were found to be necessary for the surface expression of membrane type-1 matrix metalloproteinase, thus promoting melanoma cell invasion and metastasis in vivo [15]. Tks4 plays a role in adipocyte differentiation, [18] and white and beige adipocyte biology [19]. By contrast, a role in neural-crest-derived cell type development has been attributed only to Tks5 [20,21]. Mutations in the Tks4 gene lead to a rare genetic disease called Frank-ter Haar Syndrome (FTHS) [22]. In FTHS-affected individuals, the development of several tissues is disturbed [23,24], showing that Tks4 has an effect on bone, fat, and mesenchymal stem cells [25,26]. Moreover, Tks4 has an instructive role in an epithelial mesenchymal (EMT)-like transition in cancer cells [27]. Although multiple functions of Tks4 have been described, its exact role and position in signaling pathways and the molecular mechanisms underlying its regulation remain largely unknown.

It has already been shown that Tks4 has a signal-transmitting role in EGFR signaling [28,29]. In the absence of EGF stimulation, Tks4 is mainly distributed in the cytosol. Upon EGFR activation, activated Src kinase phosphorylates Tks4 at residues Y25, Y373, and Y508 [13]. Upon phosphorylation, Tks4 translocates to the plasma membrane where it binds phosphatidylinositol lipids via its PX domain. Membrane-anchored Tks4 can then interact with other signaling molecules [17]. The cytosol-to-cell membrane translocation of signaling molecules is a widespread regulatory mechanism in signal transduction [4,30,31].

**Figure 1:**
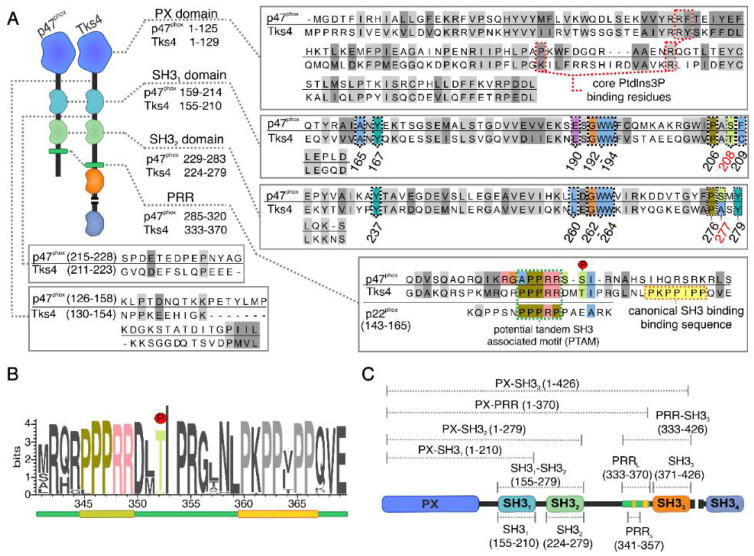
Tks4 and p47^phox^ show similarities in sequence and domain architecture. (**A**) Schematic representation of the domain architecture and sequence alignment of various regions of Tks4 and p47^phox^. Identical and similar residues are highlighted in light and dark grey, respectively. Residues comprising the canonical PtdIns(3)P-binding pockets in the PX domains are highlighted in red dashed boxes. Critical residues comprising the binding pocket of the tandem SH3 domains and the SH3_1_–SH3_2_ interface (black dashed boxes) are numbered. Ser208 and Ser277, important interacting sites in p47^phox^ (red numbers), are replaced by Thr and Ala, respectively, in Tks4. A tandem SH3-associated motif (PTAM) can be found in p47^phox^, p22^phox^, and in Tks4. However, the potential SH3 ligand found in Tks4 (yellow) is missing in p47^phox^. Only a single regulatory phosphorylation site in p47^phox^ (Ser304) seems to be conserved in Tks4 (Thr353). (**B**) Alignment of the conserved regions containing the potential tandem SH3-associated motif (PTAM) in Tks4 proteins represented by a sequence LOGO. The schematic representation of the whole region (green) including the PTAM (PPPRR, pale green) and the potential SH3-binding motif (PKPP(V/I)PP, yellow) shown below the sequence is used in all figures throughout this work. The conserved threonine residue is the only known phosphorylation site in this region. (**C**) Tks4 fragments were used in this work. Domains were color coded in all schematic representations below, as shown in this panel.

While the structural basis of the activation-induced translocation is not known in Tks4, the molecular structure, interaction partners, and regulation have already been revealed by several studies in the case of p47^phox^ [32,33,34]. In this protein, the *N*-terminal PX domain is followed by two SH3 domains (often called tandem SH3 domains) [35]. The unstructured linker region (residues 126–158) between the PX and the first SH3 domain is relatively short. The SH3 domains are connected by 14 residues. The *C*-terminal part of the molecule (residues 284–390) is unstructured, and it has an important role in the regulation of NADPH oxidase complexes [12,34]. p47^phox^ contains one proline-rich region (PRR) that serves as a binding motif for the SH3 domains of p67^phox^ [36]. The region between the second SH3 domain and the PRR is described as the autoinhibitory region (AIR) of p47^phox^. The AIR is rich in serine and arginine and contains a proline-proline-arginine (“PPR” ) core motif [12]. Based on recent studies, p47^phox^ is autoinhibited by two unique intermolecular interactions: the tandem SH3 domain binds to the AIR, and the PX domain interacts with the first SH3 domain [32,33,37]. In this closed conformation, the membrane localization of p47^phox^ and the assembly of the NADPH oxidase complex are inhibited [33]. The molecular mechanism of the tandem SH3 and AIR interaction is different from canonical SH3/PxxP binding because the AIR ligand-binding groove is formed via the conventional ligand-binding surfaces of SH3_1_ and SH3_2_ in opposing orientations [38]. In p47^phox^, SH3_1_ and SH3_2_ form a large hydrophobic binding surface that is occupied by the AIR (residues 296–305, RGAPPRRSSI) [38]. This interaction is stabilized by hydrogen bonds and salt-bridges [38]. The SH3_1_–SH3_2_ connection is also stabilized by hydrogen bonds and hydrophobic interactions [38]. The involved residues are colored in Figure 1A.

During assembly of the NADPH oxidase complex, multiple phosphorylation events on Ser residues in the AIR sequence result in the opening of the inhibited conformation of p47^phox^ [39,40]. Upon phosphorylation, p47^phox^ migrates from the cytosol to the plasma membrane, and the released SH3_1_ and SH3_2_ domains bind as a pair to an AIR-like sequence in the cytoplasmic region of p22^phox^ [12,34,35,40]. This regulatory mechanism suggests that the tandem SH3 functions as an independent module.

Alignment of the *N*-terminal regions of human Tks4 and p47^phox^ clearly shows high sequence similarity between the two proteins (Figure 1A). Most of the residues participating in the formation of the ligand-binding surface and the SH3–SH3 interface in p47^phox^ are conserved in the tandem SH3 domains of Tks4. Only two residues, Ser208 and Ser277, are replaced by Thr and Ala, respectively. Moreover, the unstructured regions between the SH3_1_ and SH3_2_ domains of the two proteins have similar lengths (Figure 1A).

The proline-proline-arginine (PPR) core motif within the AIR in p47^phox^ (and also in the binding motif of p22^phox^ [35,41]) can be identified in Tks4. This PPR motif is also called as potential tandem SH3-associated motif (PTAM) in p47^phox^-related organizer superfamily proteins. Not only the PTAM but also the flanking regions are highly conserved in Tks4 proteins (Figure 1B, Figure S1). However, these flanking regions in Tks4 and p47^phox^ are different and the distance between the SH3_2_ domain and the PTAM sequence is approximately 50 residues longer in Tks4. Moreover, another conserved potential SH3-domain-binding motif (PKPPIPP) is located adjacent to the PTAM in Tks4 (Figure 1A,B). Another important difference in the linker regions of these two proteins is that four Ser residues (303, 304, 315, and 320), whose phosphorylation has an important role in the regulation of p47^phox^, are absent in Tks4 [39,40]. There is only a single known phosphorylation site (Thr353) in close proximity of the PTAM in Tks4 (Figure 1A) [42,43,44].

Although the structural similarities between the *N*-terminal regions of Tks4 and p47^phox^ have long been recognized, whether a similar intramolecular interaction pattern and regulatory mechanism exist in Tks4 has not been investigated. The membrane localization of the protein is regulated by an EGF-dependent mechanism [28,29], suggesting the existence of inactive (cytosolic) and active (membrane-bound) conformations. Furthermore, the PX domain was found to interact with the third, but not with the first or second SH3 domains [18]. Based on these observations and the domain architecture similarities in Tks4 and p47^phox^ (Figure 1A), we hypothesized that the corresponding structural elements might be similarly responsible for the autoinhibited state in these proteins.

Here, we report the characterization of the intramolecular interactions within the *N*-terminal region of Tks4. Small angle X-ray scattering (SAXS) and protein–protein interaction assays showed that, in the closed conformation, the first two SH3 domains cooperate with each other to bind the PTAM-containing region and the third SH3 domain interacts with the PX domain. PIP strip and liposome co-sedimentation assays identified phosphatidylinositol (3)-phosphate (PtdIns(3)P) as the main lipid binding partner of the PX domain. In the autoinhibited state, PtdIns(3)P binding is blocked. Introduction of a phosphomimicking mutation into the PRR suggests a potential mechanism by which the opening of the autoinhibited state might be regulated in vivo.

## 2. Results

### 2.1. Mapping the Intermolecular Interactions in the Tks4 N Terminus

**Figure 2:**
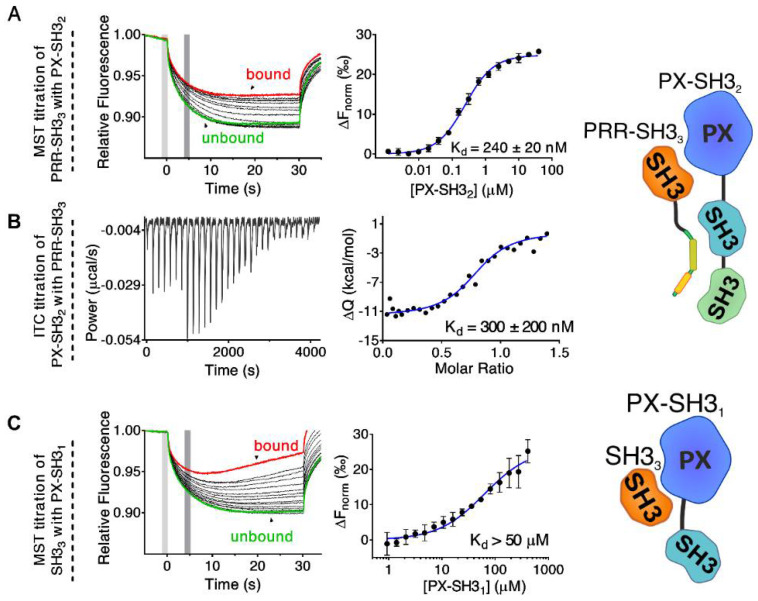
Determination of the intramolecular interactions in the Tks4 *N*-terminus via MST and ITC. Titration of PX–SH3_2_ with PRR–SH3_3_ was followed by MST (**A**) and ITC (**B**). Similar dissociation constants were observed via both methods (MST: K_d_ = 240 ± 20 nM, ITC: K_d_ = 300 ± 200 nM). The titration of PX–SH3_1_ with SH3_3_ was followed by MST (**C**). Due to the relatively weak interaction, only a lower limit was determined for the dissociation constant (K_d_ > 50 µM). Left panels: MST and ITC traces, right panels: dose-response curves. The change in the normalized fluorescence (ΔF_norm_) in the MST experiments was calculated as the ratio of the average fluorescence in the “cold” (light gray area) and “hot” states (dark gray area) of the system. MST traces corresponding to bound and unbound states of the fluorescently labeled proteins are highlighted in red and green, respectively. Dose-response data were analyzed by fitting to a quadratic equation describing a simple molecular interaction with a 1:1 stoichiometry (MST) or using the “one-set-of-independent-sites” model (ITC). Error bars depict the standard deviation of three independent measurements.

First, the *N*-terminal region of Tks4 was expressed and purified as two fragments (Figure 1C): PX–SH3_2_ (residues 1–279) and PRR–SH3_3_ (residues 333–426). Titration of fluorescently labeled PRR–SH3_3_ (see Materials and Methods) with PX–SH3_2_ was followed by MST. A relatively strong interaction with a dissociation constant (K_d_) of approximately 240 ± 20 nM was observed (Figure 2A). The reverse titration experiment (PX–SH3_2_ titrated with PRR–SH3_3_) was also performed (Figure 2B). In this case, ITC was used to follow the reaction. A similar K_d_ (300 ± 200 nM) was observed (although, the relatively weak heat changes associated with the reaction made it difficult to determine the K_d_ with high accuracy).

Binding of the PX domain to SH3_3_ has already been reported in Tks4 [18]. To understand the contribution of this interaction to the formation of the autoinhibited conformation, we titrated the fluorescently labelled SH3_3_ domain with a fragment containing the PX and SH3_1_ domains (PX–SH3_1_) and followed the reaction via MST. While the fragment corresponding to the PX domain alone could be expressed in *E. coli* cells, the purified protein was found to be unstable and precipitated slowly. By contrast, the PX–SH3_1_ construct showed no stability issues. Although the experiment clearly showed an interaction between PX–SH3_1_ and SH3_3_ (Figure 2C), the association was too weak to determine the K_d_ with high accuracy (K_d_ > 50 µM). As a control experiment, labelled SH3_3_ domain was titrated with SH3_1_. The SH3_1_ domain alone showed no binding to the SH3_3_ domain (Figure S3).

**Figure 3:**
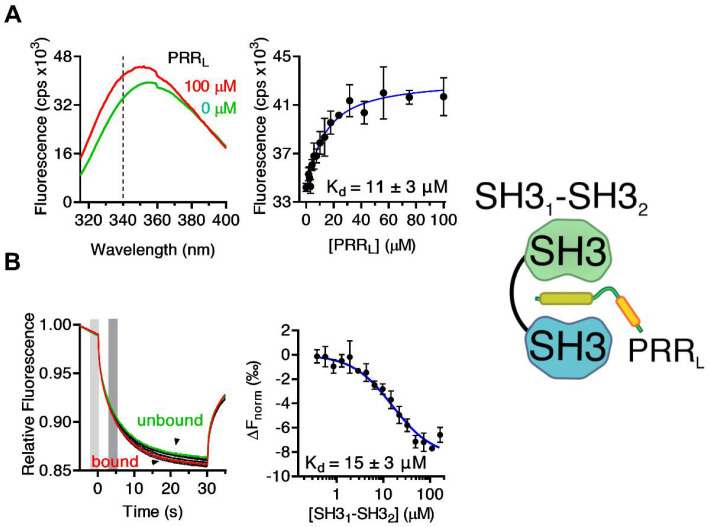
Binding of PRR_L_ to SH3_1_–SH3_2_. (**A**) Intrinsic tryptophan fluorescence emission spectra of free and PRR_L_-bound SH3_1_–SH3_2_ (left panel). In tryptophan fluorescence-based titration experiments, complex formation was followed at 340 nm (dashed line) at which the highest intensity difference was observed. A K_d_ of 24 µM is consistent with the hypothesized intramolecular binding of PRR to the tandem SH3 (SH3_1_–SH3_2_) domain of Tks4. (**B**) Binding of PRR_L_ (PTAM and PxxP are symbolized by green and yellow boxes, respectively) to SH3_1_–SH3_2_ was further validated via MST. PRR_L_ was labeled fluorescently and titrated with SH3_1_–SH3_2_. A dissociation constant of 15 µM was determined by fitting the dose-response data to a standard fitting mode derived from the law of mass action. Left panel: MST traces, right panel: dose-response curve. The change in the normalized fluorescence (ΔF_norm_) was calculated as the ratio of the average fluorescence in the “cold” (light gray area) and “hot” states (dark gray area) of the system. Traces corresponding to bound and unbound states of fluorescently labeled PRR_L_ are highlighted in red and green, respectively. Error bars depict the standard deviation of three independent measurements.

While the relatively weak interaction between PX–SH3_1_ and SH3_3_ (K_d_ > 50 µM) indicates that the association of the PX domain and the third SH3 domain might contribute to the formation of the autoinhibited conformation in full-length Tks4, the much stronger binding of PX–SH3_2_ to PRR–SH3_3_ (K_d_ ≈ 240 nM) supports the hypothesis that the PRR following the second SH3 domain might fold back and associate with SH3_1_–SH3_2_, as in p47^phox^ [12,37,38,41,45]. To test this hypothesis, the PRR_L_ fragment (residues: 333–370) containing both the PTAM and the -PxxP- motifs was titrated with SH3_1_, SH3_2_, SH3_3_, and SH3_1_–SH3_2_. Titrations were followed based on the change in the intrinsic tryptophan fluorescence emission spectra of the SH3 domains. This approach is generally used for SH3–ligand binding determination when the ligand does not contain a Trp residue [46,47,48,49,50]. A remarkable blue shift associated with an increased emission intensity was observed in the spectra of SH3_1_–SH3_2_ upon the addition of PRR_L_ (Figure 3A). No significant differences were detected in other cases (Figure S4). As the largest difference in the fluorescence intensity between the ligand-bound and free states was observed at 340 nm, we followed the titration at this wavelength, and a dissociation constant of 11 ± 3 µM was determined (Figure 3A, Table 1). Titration of a fluorescently labeled SH3_1_–SH3_2_ fragment with PRR_L_ followed by MST confirmed these results (K_d_ = 15 ± 3 µM) (Figure 3B, Table 1).

**Figure 4:**
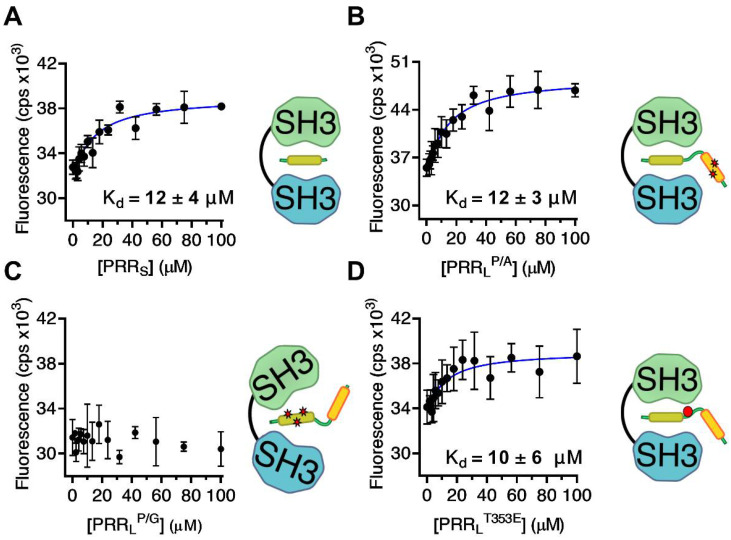
Binding of truncated and mutant PRR fragments to the SH3_1_–SH3_2_ domain of Tks4. Dissociation constants were determined via tryptophan fluorescence-based titration experiments. PRR_S_ (**A**) is a truncated form of PRR_L_. In PRR_L_^P/A^ (**B**) Pro-to-Ala mutations were introduced to disrupt the second proline-rich motif (yellow box) in PRR_L_. (See Table 1 for sequences.) The -PPPRR- potential tandem SH3-associated motif (PTAM, green box) was intact in these variants, and they showed a K_d_ similar to that of wild type PRR_L_. However, Pro-to-Gly substitutions in the PTAM core motif in PRR_L_^P/G^ (**C**) totally abolished the binding to SH3_1_–SH3_2_. The phosphomimic Thr-to-Glu mutation in PRR_L_^T353E^ (**D**) resulted only in a negligible change in the binding affinity. Error bars depict the S.D. of three independent measurements.

To identify the proline-rich binding motif within the PRR_L_ fragment that is responsible for the interaction with SH3_1_–SH3_2_, a truncated version of PRR_L_ containing only the PTAM (PRR_S_) was synthesized (Table 1). Additionally, two PRR_L_ mutants were expressed and purified: PRR_L_^P/A^ and PRR_L_^P/G^ (Table 1). In PRR_L_^P/A^, two central proline-to-alanine substitutions were made in the canonical SH3-binding motif residues: -PKPPIPP-, whereas in PRR_L_^P/G^, three proline-to-glycine substitutions were made in the PTAM (residues: -PPPRR-). Dissociation constants were determined via Trp-fluorescence-based titration experiments (Figure 4, Figure S5). Both PRR_S_ and PRR_L_^P/A^ could bind SH3_1_–SH3_2_ with a K_d_ similar to that of the wild-type PRR_L_ fragment (Figure 4A,B, Table 1). However, lack of the core motif in PRR_L_^P/G^ resulted in total loss of ligand binding (Figure 4C, Table 1). These results strongly suggest that, in full-length Tks4, the core binding motif of SH3_1_–SH3_2_ is indeed the -PPPRR- sequence. The flanking regions, such as the -PKPPIPP- motif, make no or negligible contribution to binding.

Previous studies have shown that phosphorylation of Ser303 and Ser304 plays an important role in the regulation of p47^phox^ via the disruption of its intramolecular interactions [39,40]. Tks4 contains a single threonine residue (Thr353) in a homologous position (Figure 1). Phosphorylation of Thr353 has been reported in multiple studies [42,43,44]. We hypothesized that phosphorylation of Thr353 in Tks4 might have a regulatory role similar to that of Ser303 and Ser304 in p47^phox^ by disrupting the autoinhibited conformation. Interestingly, titration of SH3_1_–SH3_2_ with PRR_L_^T353E^, a phosphomimetic mutant of PRR_L_, yielded a K_d_ close to the dissociation constant of wild type PRR_L_ (Figure 4D, Table 1). The unchanged dissociation constant strongly suggests that phosphorylation of Thr353 does not influence the binding between the tandem SH3_1_–SH3_2_ domain and the proline-rich region in full-length Tks4.

### 2.2. Confirmation of the Closed Conformation of the Tks4 N-Terminal Region via SAXS Analysis

**Figure 5:**
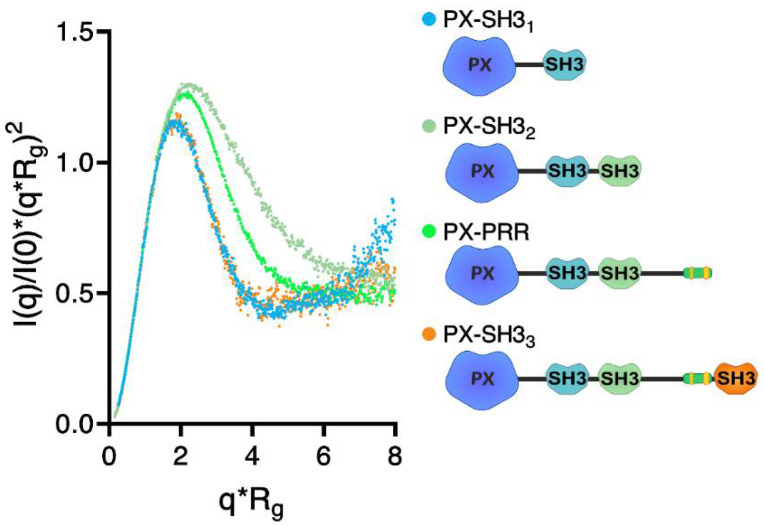
Normalized Kratky plot of the Tks4 constructs. The bell-shaped profiles of PX–SH3_1_ and PX–SH3_3_ indicate a compact, globular-like state. The profiles of PX–SH3_2_ and PX–PRR are consistent with a more extended and flexible state. Despite being a long, unstructured linker region, the presence of the PRR resulted in increased compactness (compare PX–SH3_2_ and PX–PRR). Addition of the SH3_3_ domain further increased the compactness.

To gain more insight into the formation of the autoinhibited conformation of Tks4, four truncated constructs, i.e., PX–SH3_1_, PX–SH3_2_, PX–PRR, and PX–SH3_3_, were generated (Figure 1C). Purified samples of these Tks4 fragments were analyzed via SAXS to obtain structural information. As expected, the increasing molecular weight of the fragments was accompanied by increasing Porod volumes (Table 2). Compared with PX–SH3_1_, the presence of the second SH3 domain in PX–SH3_2_ resulted in an increased radius of gyration (R_g_). However, further extension of PX–SH3_2_ with a long, disordered region in PX–PRR did not affect the R_g_. Moreover, the presence of the third SH3 domain in PX–SH3_3_ resulted in a decreased R_g_ (Table 2). The maximal length (D_max_) of the constructs followed a similar pattern, although the highest D_max_ was observed in the case of PX–PRR (Table 2). A normalized Kratky plot for PX–SH3_1_ (Figure 5) showed a bell-shaped profile consistent with a relatively compact, globular-like state. Extension of PX–SH3_1_ with the second SH3 domain in PX–SH3_2_ dramatically altered this bell-like profile, indicating an extended, more flexible conformation. Interestingly, further extension with the PRR region in PX–PRR resulted in a profile consistent with a less flexible state. Moreover, introduction of the third SH3 domain in PX–SH3_3_ yielded a profile almost identical to the profile of PX–SH3_1_ (Figure 5). These data show that both the PRR and the third SH3 domain fold back and interact with the PX–SH3_1_–SH3_2_ region, yielding a relatively compact conformation.

**Figure 6:**
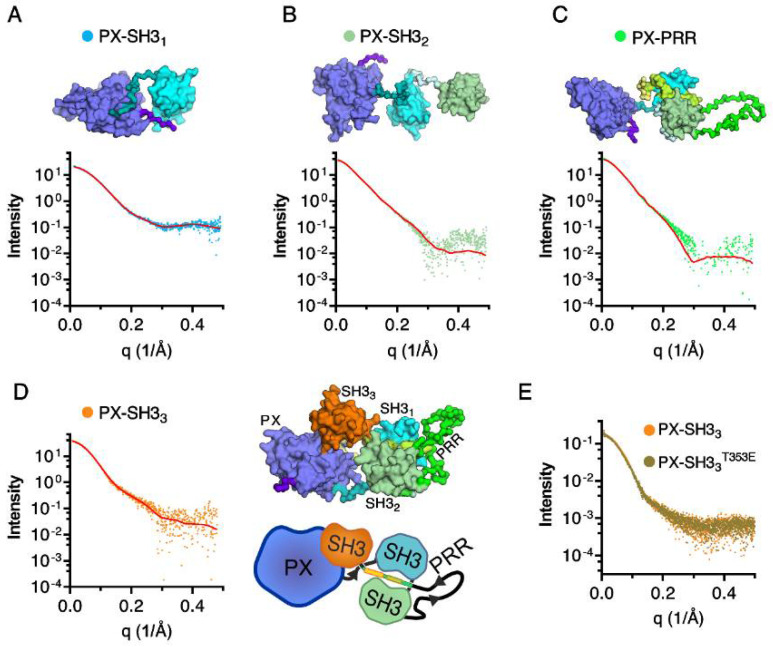
SAXS-based rigid body modeling of Tks4 fragments. Experimental scattering curves and computed scattering intensity generated by CORAL for the Tks4 fragments PX–SH3_1_ (**A**), PX–SH3_2_ (**B**), PX–PRR (**C**), and PX–SH3_3_ (**D**) are shown in black and red, respectively. Atomic models were generated based on the 1UEC, 1WLP, and 1GD5 structures using MODELLER. Domains and linker regions are shown in different colors to guide the eyes (PX: blue, SH3_1_: cyan, SH3_2_: light green, PRR: lime and green, SH3_3_: orange.) The schematic representation of the autoinhibited structure of the full *N*-terminal region is shown in panel D (**E**) Scattering curves of the T353 mutant and wild-type PX–SH3_3_ constructs.

Rigid body modeling of all Tks4 fragments was performed via CORAL (Figure 6). The overlap between the model-based theoretical scattering intensity and the experimental scattering data showed that the generated models were consistent with the experimental data (Figure 6). Chi (χ) values indicate good fits, particularly in the case of the shortest (PX–SH3_1_) and longest (PX–SH3_3_) fragments (Table 2). The rigid body models indicate no interaction between the PX domain and the first or the second SH3 domains, but show a close proximity between the PX domain and third SH3 domain.

Finally, the potential effect of phosphorylation in the PRR on the conformation of PX–SH3_3_ was investigated by using the PX–SH3_3_^T353E^ mutant. The experimental scattering curves of PX–SH3_3_ and PX–SH3_3_^T353E^ (Figure 6E) and all of the structural parameters (Table 2) were nearly identical, indicating that phosphorylation of Thr353 is most likely not the regulatory mechanism responsible for the opening of the closed conformation of Tks4.

### 2.3. Lipid Binding Assays

PX domains are responsible for the attachment of proteins to membranes by binding phosphoinositide lipids [8,9]. In p47^phox^, lipid binding by the PX domain is inhibited by autoregulatory intramolecular interactions [40,51]. Based on domain architecture similarities and similar intramolecular interaction patterns observed in Tks4, we hypothesized that the closed conformation of the *N*-terminus results in inhibition of the PX domain via a similar mechanism. To test this hypothesis, the binding specificity and relative affinity of hexahistidine-tagged (His_6_-) Tks4 fragments were determined via protein–lipid overlay and liposome co-sedimentation assays.

**Figure 7:**
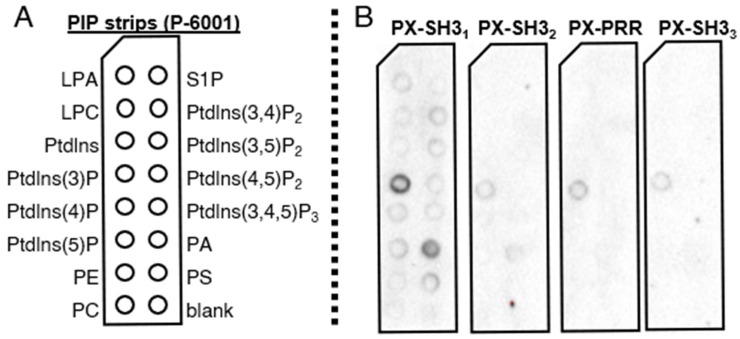
PX domain-containing Tks4 fragments bind phosphatidylinositol (3)-phosphate and phosphatidic acid in a protein–lipid overlay (“PIP strip”) assay. (**A**) Schematic representation of the PIP strip membrane. LPA: lysophosphatidic acid, LPC: lysophosphatidylcholine, PtdIns: phosphatidylinositol, PtdIns(3)P: phosphatidylinositol (3)-phosphate, PtdIns(4)P: phosphatidylinositol (4)-phosphate, PtdIns(5)P: phosphatidylinositol (5)-phosphate, PE: phosphatidylethanolamine, PC: phosphatidylcholine, S1P: sphingosine 1-phosphate, PtdIns(3,4)P_2_: phosphatidylinositol (3,4)-bisphosphate, PtdIns(3,5)P_2_: phosphatidylinositol (3,5)-bisphosphate, PtdIns(4,5)P_2_: phosphatidylinositol (4,5)-bisphosphate, PtdIns(3,4,5)P_3_: phosphatidylinositol (3,4,5)-trisphosphate, PA: phosphatidic acid, PS: phosphatidylserine. (**B**) The His_6_–PX–SH3_1_ and His_6_–PX–SH3_2_ Tks4 fragments bound both PtdIns(3)P and PA, while His_6_–PX–PRR and His_6_–PX–SH3_3_ bound PtdIns(3)P only.

First, purified His_6_–PX–SH3_1_, His_6_–PX–SH3_2_, His_6_–PX–PRR, and His_6_–PX–SH3_3_ were incubated with 15 different phospholipids spotted onto the surface of a nitrocellulose membrane (“PIP strip” assay, Echelon Biosciences). Following three washing steps, the membranes were incubated with mouse anti-His_6_ and anti-mouse IgG-HRP antibodies, and bound immunocomplexes were detected via enhanced chemiluminescence (Figure 7). His_6_–PX–SH3_1_ and His_6_–PX–SH3_2_ exhibited affinity mainly for Ptdlns(3)P (phosphatidylinositol (3)-phosphate) and PA (phosphatidic acid). The longer Tks4 fragments His_6_–PX–PRR and His_6_–PX–SH3_3_ showed an affinity for Ptdlns(3)P only (Figure 7).

It is important to note that the PIP strip assay provides only a qualitative estimate of the lipid binding specificity. Quantitative comparisons of lipid binding affinities between different Tks4 fragments are not possible. The extensive signal amplification, which allows the detection of relatively weak protein–lipid interactions, may explain why all of the Tks4 fragments interacted with Ptdlns(3)P in this assay. In His_6_–PX–PRR and His_6_–PX–SH3_3_, autoinhibition may have reduced lipid binding; however, autoinhibition is a conformational equilibrium between the autoinhibited (closed) and active (opened) states. Even if the active pool of the protein is present in relatively low quantities, it can still interact with the lipid surface and give an observable signal if substantial amplification is applied. Altogether, these results suggest that the main lipid ligand of the PX domain is Ptdlns(3)P, although there might be some cross-reactivity with PA.

**Figure 8:**
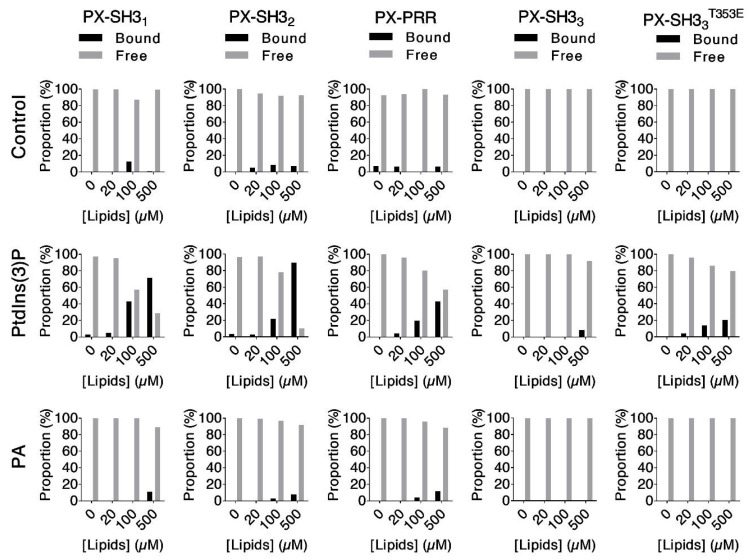
Liposome co-sedimentation assays: The Tks4 fragments PX–SH3_1_, PX–SH3_2_, PX–PRR, and PX–SH3_3_ at a final concentration of 1 µM were titrated with phospholipid-free control liposomes (top panels), liposomes containing 5% (mol/mol) PtdIns(3)P (middle panels), or liposomes containing 5% (mol/mol) PA (bottom panels). Liposomes with bound proteins were separated from the soluble fraction via centrifugation. Samples were analyzed via SDS-PAGE and Coomassie staining (Figure S6). The percentages of fragments recovered in the bound (black) and free (gray) fractions were determined via gel densitometry. Only PtdIns(3)P-containing liposomes showed concentration-dependent association with Tks4 fragments. This effect was reduced in the case of PX–PRR and almost totally abolished in the case of PX–SH3_3_, showing that both the PRR and the third SH3 domain have autoinhibitory roles in Tks4. Although the PX–SH3_3_^T353E^ mutant showed slightly increased PtdIns(3)P liposome binding, it seems very unlikely that the phosphorylation of Thr353 could be the regulatory mechanism responsible for Tks4 activation in vivo. None of the Tks4 fragments associated with control or PA-containing liposomes.

Neither Ptdlns(3)P nor PA form biological membranes alone in a cell. Moreover, the curvature and various components of a real biological membrane may change the accessibility and the interaction of these lipids with their protein partners. Therefore, we re-tested the binding of our Tks4 fragments to Ptdlns(3)P and PA in liposome co-sedimentation assays. In these experiments, Ptdlns(3)P and PA were mixed with different lipids (see Materials and Methods) to form liposomes. Tks4 fragments at a final concentration of 1 µM (PX–SH3_1_, PX–SH3_2_, PX–PRR, and PX–SH3_3_) were titrated with liposome solutions. Ptdlns(3)P or PA presented 5% of the total lipid pool, and the final total lipid concentrations were 0 µM, 20 µM, 100 µM, and 500 µM in all titration experiments. Liposome-bound proteins were separated from the free fraction via centrifugation. Protein samples were analyzed via SDS-PAGE and Coomassie staining. Quantitation was performed via densitometry (Figure 8, Figure S6). This experimental setup more closely mimics the environment in which the lipids of interest exist in vivo and allows semi-quantitative comparisons of the relative lipid binding affinities of the Tks4 fragments.

Concentration-dependent depletion of the free (soluble) protein pool was observed in the case of PX–SH3_1_ when titrated with liposomes containing Ptdlns(3)P. PX–SH3_2_ clearly showed the same effect. The presence of the PRR in PX–PRR reduced Ptdlns(3)P binding. The addition of the third SH3 domain (in PX–SH3_3_) completely abolished the interaction. None of the Tks4 fragments interacted with PA-containing liposomes (Figure 8). These results indicate that Ptdlns(3)P, not PA, is the most likely lipid target of the PX domain in vivo. Lipid binding by the PX domain is regulated by intramolecular interactions. Unlike in p47^phox^ [33,45,52], both the third SH3 domain and the PRR are required for effective autoinhibition in Tks4 (there is no third SH3 domain in p47^phox^).

Finally, we investigated the hypothesized regulatory role of Thr353 via liposome co-sedimentation assays by using the phosphomimic mutant PX–SH3_3_^T353E^. Compared with the wild type PX–SH3_3_ fragment, PX–SH3_3_^T353E^ showed only slightly increased PtdIns(3)P binding. This small effect size suggests that Thr353 phosphorylation is most likely not the regulatory mechanism responsible for the closed-to-open transition of Tks4 in vivo.

## 3. Discussion

The *N*-terminal region of Tks4 closely resembles the structure of p47^phox^, an evolutionarily-related protein in which the same domains and linear motifs exist in the same relative order (Figure 1A). In this work, we hypothesized that Tks4 is regulated by autoinhibitory intramolecular interactions similar to those observed in p47^phox^ [12,33,52]. A straightforward way to prove this concept and to shed light on the atomic-level details of the mechanism would be to crystallize the *N*-terminal region of Tks4 and to solve the structure via X-ray diffraction. Unfortunately, all our attempts to crystallize various Tks4 fragments failed (data not shown), most probably due to the presence of the disordered linker regions connecting the PX and SH3 domains. Therefore, we applied multiple protein–protein interaction assays to identify interactions between various Tks4 fragments. Two main interaction sites were identified: (1) between the PTAM and the SH3_1_–SH3_2_ domains; and (2) between the PX domain and the SH3_3_ domain. Interestingly, neither SH3_1_ nor SH3_2_ bound the PTAM alone. The presence of both domains is necessary for a detectable interaction. The regular -PxPPxPP- SH3-binding motif located close to the PTAM (Figure 1A) showed no interaction with SH3_1_–SH3_2_.

In addition to autoinhibition, tandem SH3 domains in other members of the p47^phox^-related organizer superfamily are known to have a role in partner protein recognition and binding. For example, the interaction of Tks5 and Sos1 is mediated by the SH3_1_–SH3_2_ domains in Tks5 [53]. The binding of Dynamin, IRTKS, and p22^phox^ also requires the presence of SH3_1_–SH3_2_ domains [54,55]. These observations suggest that the tandem SH3 domains may also have a dual role in Tks4, i.e., autoinhibition (which requires intramolecular interaction with the PRR) and partner binding. Partner binding might activate Tks4 since the binding groove in the SH3_1_–SH3_2_ domain cannot be accessible to the PRR while a partner occupies it. Alternatively, an activation signal (such as phosphorylation) might first be required to disrupt the interaction between the SH3_1_–SH3_2_ domains and the PRR. Such a signal might lead to simultaneous activation of the PX and SH3_1_–SH3_2_ domains, making them accessible to their lipid and protein binding partners, respectively.

In the case of p47^phox^, Marcoux et al. applied SAXS and found that the PX domain was located in the proximity of the first SH3 domain [33]. However, it was not clear whether the PX domain interacts directly with the SH3_1_ domain or whether it is maintained only in close proximity by a structured linker [33]. Surprisingly, the SAXS data presented in this work indicated no direct interaction (physical binding) between the PX domain and the SH3_1_–SH3_2_–PRR (containing the PTAM motif) region of Tks4. However, the intramolecular binding of the PTAM to SH3_1_–SH3_2_ can still reduce the ability of the PX domain to bind PtdIns(3)P (see Figure 8). We hypothesize that the formation of this intramolecular interaction leads to the positioning of the second SH3 domain closer to the PX domain (compare the models shown in Figure 6B,C) where it can interfere with the binding of PtdIns(3)P simply by occupying too much space around the lipid binding site, thereby blocking the ability of the PX domain to access membrane surfaces rich in PtdIns(3)P.

An interaction between the PX and SH3_3_ domains in Tks4 has been observed by Hishida et al. [18]. Our MST measurements confirmed these results: the SH3_3_ domain showed a weak interaction with the PX domain. This observation suggests that the Tks4 protein has an extra intramolecular connection in addition to those present in p47^phox^. It should be noted, however, that the apparent K_d_ values of the separate domains most likely do not reflect the true strength of the interaction when these domains are connected in the intact molecule. In that situation, the two domains remain in close proximity of each other, resulting in high apparent local concentrations and, eventually, a much stronger interaction. Therefore, even if only relatively weak interactions were identified in this work, we can still conclude that these interactions might reflect an effective regulatory mechanism. This conclusion is supported by the fact that PX–SH3_3_ has a folded, compact structure according to our SAXS experiments (Table 2, Figure 5 and Figure 6). Furthermore, the interaction between the tandem SH3 domains and the PRR could only reduce liposome binding in co-sedimentation assays. For full inhibition, the presence of the SH3_3_ domain was essential (Figure 8).

Intramolecular interactions always imply the possibility of protein dimerization or oligomerization. Our SAXS analysis has indeed revealed some (mostly mild) concentration-dependent aggregation (see Figure S2). However, the proximity of any two interacting domains within the same protein always favors intramolecular interactions due to the high apparent local concentrations (unless the linker is too short or too long, such that the interaction is sterically hindered or the two domains are practically independent, respectively). Although we cannot exclude the possibility of homodimeriztion in the case of Tks4 in vivo, our results are mainly consistent with the intramolecular interactions hypothesis and clearly show that these interactions are crucial in the regulation of the molecule.

While our protein–lipid overlay assay showed that the PX domain can selectively bind PtdIns(3)P and PA, the liposome co-sedimentation assay confirmed only the interaction with PtdIns(3)P. In this assay, the environment of the lipids (liposomal membrane) closely resembles that found in the cell membrane. This is a better model system than the PIP strip, where lipids are loaded onto a flat, artificial surface. Therefore, the liposome co-sedimentation assay yields more reliable results that better reflect the in vivo conditions. Interestingly, some studies have reported interactions between the PX domain of Tks4 and other phosphoinositide lipids using dot-blot assays [13,18,29,56]. All these studies used GST-tagged PX domains. The dimerization of GST is known to alter the binding profile by increasing the apparent binding affinity of a given domain [57]. In our experiments, the protein fragments were monomeric. This difference might explain why we only observed an interaction with PtdIns(3)P, which is most likely the strongest in vivo-relevant binding partner of the Tks4 PX domain. Interestingly, it has been observed that the PX domain of Tks5 also interacts with PtdIns(3)P [58,59] and that truncated forms of these proteins (containing the PX domain only) localize to punctate structures in cells [18,59]. PtdIns(3)P is present predominantly in endosomes [60,61] and punctate staining is typically seen in association with endosomal membranes [59]. However, the full-length proteins localized mostly to the cytoplasm [18,59], also suggesting that the PX domain–lipid interaction is probably inhibited in the full-length proteins via intramolecular interactions.

We attempted to identify the mechanism by which the closed conformation can be disrupted in Tks4. p47^phox^ undergoes conformational changes upon phosphorylation of multiple Ser residues (Ser303, Ser304, Ser315, Ser320, and Ser328) in the PRR. It is believed that these phosphorylation events induce the opening of p47^phox^ [39,40,41,62]. Following this conformational change, the tandem SH3 domains become unoccupied and thus available to interact with p22^phox^ [37,40,63]. Along with this, the locked PX domain is released, subsequently anchoring the protein to the membrane [51]. In Tks4, Thr353 is the only known phosphorylation site close to the PTAM [42,43,44]. Therefore, we analyzed the structure and lipid-binding properties of the phosphomimic mutant T353E via SAXS and liposome co-sedimentation assays, respectively. No major conformational changes were observed via SAXS. Moreover, the co-sedimentation assays showed only partial restoration of the interaction with PtdIns(3)P-containing liposomes. Although the structure and intramolecular interactions are similar in Tks4 and p47^phox^, it seems that the opened-to-closed conformational switch is regulated differently. It is possible that the relevant Ser/Thr phosphorylation sites in Tks4 (if any) have not been identified yet. However, it is known that Src tyrosine kinase phosphorylates Tks4 at Tyr25 and Tyr373 (in the PX and SH3_3_ domains, respectively). These events are important in the activation (and subsequent membrane translocation) of the molecule, which results in the assembly of a signaling complex (i.e., binding between Src and EGFR) [13,28]. It is possible that tyrosine phosphorylation results in the disruption of inhibitory intramolecular interactions and induces conformational changes in Tks4, similar to those observed in p47^phox^ upon activation.

In summary, the intramolecular interaction between tandem SH3 domains and the PTAM motif, and the interaction between the PX and SH3_3_ domains lead to a compact packaging of the *N*-terminal region of Tks4. In this closed conformation, the ability of the PX domain to bind PtdIns(3)P in biological membranes is blocked. Phosphorylation of Thr353 does not lead to the opening of the closed conformation and can only partially restore PtdIns(3)P binding. Further studies are needed to evaluate other potential regulatory mechanisms, such as the phosphorylation of tyrosine residues by Src kinase.

## 4. Materials and Methods

### 4.1. DNA Constructs and Protein Expression

The expression vector harboring the human Tks4 gene (Uniprot accession number: A1X283) used in our experiments was described previously [56]. DNA sequences of the recombinant Tks4 fragments (PX–SH3_1_: residues 1–210, PX–SH3_2_: residues 1–279, PX–PRR: residues 1–370, PX–SH3_3_: residues 1–426, SH3_1_–SH3_2_: residues 155–279, PRR–SH3_3_: residues 333–426, SH3_1_: residues 155–210, SH3_2_: residues 224–279, SH3_3_: residues 371–426, PRR_L_: residues 333–370) were amplified by PCR and subcloned into a modified pET vector encoding an *N*-terminal His-tag followed by a TEV protease recognition site. All constructs were verified by DNA sequencing (Eurofins Genomics). The TEV protease gene cloned into a bacterial expression vector was a kind gift from Dr. László Nyitray. All proteins were expressed in *E. coli Rosetta pLysS* bacteria (Novagen) and purified as previously described [46]. In the case of PRR_L_ and its variants (PRR_L_ ^P/G^, PRR_L_ ^P/A^, PRR_L_ ^T353E^), nickel-affinity chromatography [46] was followed by a RP-HPLC purification step using a ReproSil 300 C18; 5 µm; 250 × 10 mm column (Maisch). The purity and mass of the peptides were verified using a Shimadzu LC-MS 2020 instrument.

### 4.2. Synthetic Peptides

The PRR_S_ peptide was chemically synthesized on an automated PSE Peptide Synthesizer (Protein Technologies, Tucson, AZ, USA) with a standard Fmoc/tBu SPPS strategy. The peptide was purified via RP-HPLC using a ReproSil 300 C18; 5 µm; 250 × 10 mm column (Maisch). The purity and mass of the peptides were verified using a Shimadzu LC-MS 2020 instrument.

### 4.3. Fluorescence Spectroscopy-Based Titrations

Titration of the SH3_1_–SH3_2_ (“tandem SH3”) fragment by wild type, mutant, and truncated PRR fragments (PRR_L_, PRR_S_, PRR_L_^P/G^, PRR_L_^P/A^, an PRR_L_^T353E^) was followed by the detection of the intrinsic tryptophan fluorescence signal. Measurements were performed in 384-well GreinerBio microplates (Ref.: 781076) in 50 µL final volumes. Prior to the measurements, protein samples were dialyzed against PBS (phosphate-buffered saline) supplemented with 0.5 mM TCEP and 0.05% TWEEN 20. Fifteen-step serial 3:4 dilutions of the PRR fragments were prepared and mixed with SH3_1_–SH3_2_ stock solutions to yield a constant final SH3_1_–SH3_2_ concentration of 5 µM for all experiments. The final concentrations of the PRR fragments varied between 1.8 µM and 100 µM. The emission spectra of all samples were recorded between 315–400 nm in 1-nm steps using a PerkinElmer EnSpire Plate Reader. Selective excitation of tryptophane residues was achieved by setting the excitation monochromator to 295 nm. Titrations were followed at 340 nm. The average signal intensity and standard deviation were calculated from three independent experiments. Dose-response data were analyzed by using a simple 1:1 binding equilibrium model.

### 4.4. Isothermal Titration Calorimetry (ITC)

The affinity of PX–SH3_2_ to PRR–SH3_3_ was measured using a MicroCal ITC200 instrument at 25 °C in PBS freshly supplemented with 0.5 mM TCEP. The concentration of PX–SH3_2_ was 20 μM in the measuring cell, and the concentration of the PRR–SH3_3_ domain was 250 μM in the syringe. Data were evaluated by the AFFINImeter ITC software utilizing the “one set of independent binding sites” model [64].

### 4.5. Microscale Thermophoresis (MST)

The PRR–SH3_3_, SH3_1_–SH3_2_, and SH3_3_ constructs were fluorescently labeled with Nanotemper NT-647 amine-reactive dye (NanoTemper Technologies, München, Germany) according to the manufacturer’s instructions. The PRR–SH3_3_, SH3_1_–SH3_2_ and SH3_3_ domains were titrated with PX–SH3_2_, PRR_L_, and PX–SH3_1_, respectively. All measurements were performed in PBS supplemented with 0.5 mM TCEP and 0.05% TWEEN 20 by using Monolith NT.115 Premium Capillaries (NanoTemper Technologies MO-k025) and a NanoTemper Monolith NT.115 instrument. Sixteen-step serial 2:3 dilutions of PX–SH3_1_ and PRR_L_ were prepared following the addition of the labeled ligand solutions, thus yielding final starting concentrations of 412 μM and 166 μM of PX–SH3_1_ and PRR_L_ in the titrations, respectively. In the case of PX–SH3_2_, sixteen-step serial 1:2 dilutions and a starting concentration of 40 µM were used. The concentrations of the labeled ligands were kept constant (20 nM) in all experiments. All binding assays were performed in triplicate. MST traces were recorded at room temperature. The excitation LED was used at 50% power and the IR laser power was set to 20%. Data evaluation was performed with the MO Affinity Analysis 2.3 software. Dose response curves were analyzed by using the standard K_d_ Fit Model.

### 4.6. Small Angle X-ray Scattering (SAXS) Experiments

SAXS measurements were performed using the BM29 beamline at ESRF or the P12 beamline at EMBL-Hamburg (PETRA). Data were analyzed using the ATSAS 3.0 software package [65]. Each construct was measured over a dilution series containing four different concentrations. Merging of the dilution series and primary data analysis was performed in PRIMUS [65]. Mostly minor concentration effects were observed during the measurements, which were excluded via manual merging (Figure S2). Rigid body modeling was performed in CORAL using default parameters without any symmetry constraint (P1 symmetry), for each dataset models were obtained from five independent runs giving similar structural results [65]. During rigid body modeling, rigid bodies were connected by flexible linkers allowing the in silico explorations of structural conformations. In the case of PX–PRR and PX–SH3_3_ constructs, the PRR and two SH3 domains complex were paired together in CORAL runs to treat them as one rigid body. Atomic models used in the CORAL modeling were generated with homology modeling based on the 1UEC (SH3 domain), 1WLP (PRR and SH3 domains complex), and 1GD5 (PX domain) structures using Modeller [66].

### 4.7. Protein–Lipid Overlay Assays (PIP Strip)

Overlay assays were carried out using His-tagged fragments of Tks4 (PX–SH3_1_, PX–SH3_2_, PX–PRR, and PX–SH3_3_). PIP strips were purchased from Echelon (catalog number: P-6001). All experiments were carried out according to the manufacturer’s instructions. Briefly, the strips were blocked in TBS-T (0.1% *v*/*v* TWEEN 20) with 3% fatty acid-free BSA (Sigma-Aldrich, St. Louis, MO, USA, A-6003) for 1 h at ambient temperature. The membrane was incubated with 10 ug/mL of the given protein for 1 h at room temperature, and then washed three times over 30 min with gentle agitation. The bound protein was detected via a 1-h incubation with anti-His antibody (Millipore, Burlington, MA, USA, 05-949). Following a washing step, the membranes were incubated with anti-mouse IgG-HRP (Sigma-Aldrich A-9044) for 1 h prior to detection of bound proteins using enhanced chemiluminescence.

### 4.8. Preparation of PM-Mimetic Vesicles

Lipids were purchased from Avanti Polar Lipids with the following catalog numbers: POPC (1-palmitoyl-2-oleoyl-glycero-3-phosphocholine, 850457); DOPE (1,2-dioleoyl-sn-glycero-3-phosphoethanolamine, 850725); POPS (1-palmitoyl-2-oleoyl-sn-glycero-3-phospho-L-serine, 840034); cholesterol (700000); PI (L-α-phosphatidylinositol, 840042); PA (L-α-phosphatidic acid, 840101); and PtdIns(3)P (1,2-dioctanoyl-sn-glycero-3-(phosphoinositol-3-phosphate, 850187). Control liposome samples were prepared by mixing the following lipids: 13% (mol/mol) POPC, 35% (mol/mol) DOPE, 22% (mol/mol) POPS, 22% (mol/mol) cholesterol, and 8% (mol/mol) PI. The PtdIns(3)P and PA liposomes were prepared similarly, but the concentrations of POPC and cholesterol were reduced to 10% and 20% (mol/mol), respectively, and 5 mol% PtdIns(3)P or 5 mol% PA were added to the lipid mixture. Stock solutions were prepared in methanol at a concentration of 10 mM. Prior to the experiments, the appropriate amounts of lipids were dried under a stream of N_2_-gas in glass vials. Next, the samples were further dried in vacuo for at least 2 h. Liposomes were prepared by dissolving the dried lipid samples in assay buffer by alternated vortexing and sonication for 5 min. The freshly prepared liposomes were centrifugated at 100,000× *g* for 1 h at 4 °C, and the pellets were reconstituted in PBS containing 0.1 mM TCEP. The final total lipid concentration was 2 mM.

### 4.9. Liposome Co-Sedimentation Assays

The freshly prepared liposome solutions were diluted to final total lipid concentrations of 0.01 mM, 0.1 mM, and 0.5 mM in assay buffer (PBS containing 0.1 mM TCEP). Solutions of the Tks4 fragments (PX–SH3_1_, PX–SH3_2_, PX–PRR, PX–SH3_3_, and PX–SH3_3_^T353E^) were added to the liposomes to yield a final protein concentration of 1 µM in all cases. Mixtures were incubated on ice for 30 min. Soluble and liposome-bound proteins were separated via centrifugation in a TL-100 ultracentrifuge (Beckman) using a TLA100.3 rotor (30 min, 100,000× *g*, 4 °C). The supernatants and pellets were collected and analyzed via SDS-PAGE. Bands were quantitated via densitometry using ImageJ [67].

## Figures and Tables

**Table 1:** Binding of the wild-type and mutant variants of PRR to SH3_1_–SH3_2_. Point mutations are denoted by red letters. Dissociation constants were determined by Trp-fluorescence-based titrations (FL) or microscale thermophoresis (MST). PRR_L_, PRR_L_^P/G^, PRR_L_^P/A^, and PRR_L_^T353E^ were expressed in *E. coli*. In these fragments, four residues at the *N*-terminus (GSHM) are cloning artifacts. PRR_S_ was a synthetic peptide. Errors represent the S.E. of fitting.

Tks4 Fragment	Sequence	K_d_ (µM)	Method
PRR_L_	GSHMGDAKQRSPKMRQRPPPRRDMTIPRGLNLPKPPIPPQVE	15 ± 3	MST
		11 ± 3	FL
PRR_S_	KMRQRPPPRRDMTIPRG	12 ± 4	FL
PRR_L_^P/G^	GSHMGDAKQRSPKMRQRGGGRRDMTIPRGLNLPKPPIPPQVE	-	FL
PRR_L_^P/A^	GSHMGDAKQRSPKMRQRPPPRRDMTIPRGLNLPKAAIPPQVE	12 ± 3	FL
PRR_L_^T353E^	GSHMGDAKQRSPKMRQRPPPRRDMEIPRGLNLPKPPIPPQVE	10 ± 6	FL

**Table 2:** Structural parameters determined via SAXS. MW: calculated molecular weight, R_g_: radius of gyration, D_max_: maximal length, V_P_: Porod volume, χ: chi value (CORAL modeling). Estimated parameters are shown with their respective standard errors.

Tks4 Fragment	MW (kDa)	R_g_ (nm)	D_max_ (nm)	V_P_ (nm^3^)	χ (CORAL fit)
PX–SH3_1_	24.6	2.14 ± 0.05	6.3 ± 0.3	39.1	0.74
PX–SH3_2_	33	3.04 ± 0.16	9.7 ± 0.4	63.5	1.39
PX–PRR	42.6	3.05 ± 0.28	10.3 ± 0.2	80.1	1.8
PX–SH3_3_	49	2.86 ± 0.05	9.2 ± 0.3	83.3	0.66
PX–SH3_3_^T353E^	49	2.85 ± 0.09	9.3 ± 0.3	84.7	-

## Supplementary Materials

The following are available online at https://www.mdpi.com/article/10.3390/ijms22158103/s1.

## Abbreviations

TKS4	Tyrosine kinase substrate with 4 SH3
EGF	epidermal growth factor
PX	Phox homolog
SH3	Src homolog 3
PRR	proline-rich region
MST	Microscale thermophoresis
ITC	Isothermal titration calorimetry
SAXS	Small Angle X-ray Scattering
PtdIns(3)P	Phosphatidylinositol-3-phosphate
TKS5	Tyrosine kinase substrate with 5 SH3
FTHS	Frank-ter Haar Syndrome
EMT	epithelial mesenchymal transition
AIR	autoinhibitory region
PTAM	potential tandem SH3-associated motif
PA	Phosphatidic acid
TCEP	Tris(2-carboxyethyl)phosphine
