# Supplementary material for: Characterization of the Intramolecular Interactions and Regulatory Mechanisms of the Scaffold Protein Tks4
Source: Int J Mol Sci. 2021 Jul 28;22(15):8103. doi: 10.3390/ijms22158103 (PMC8348221; doi:10.3390/ijms22158103)
Supplement: Supplementary file 1 [file ijms-22-08103-s001.zip › ijms-1296800-supplementary.pdf]

International Journal of *Molecular Sciences*

MDPI

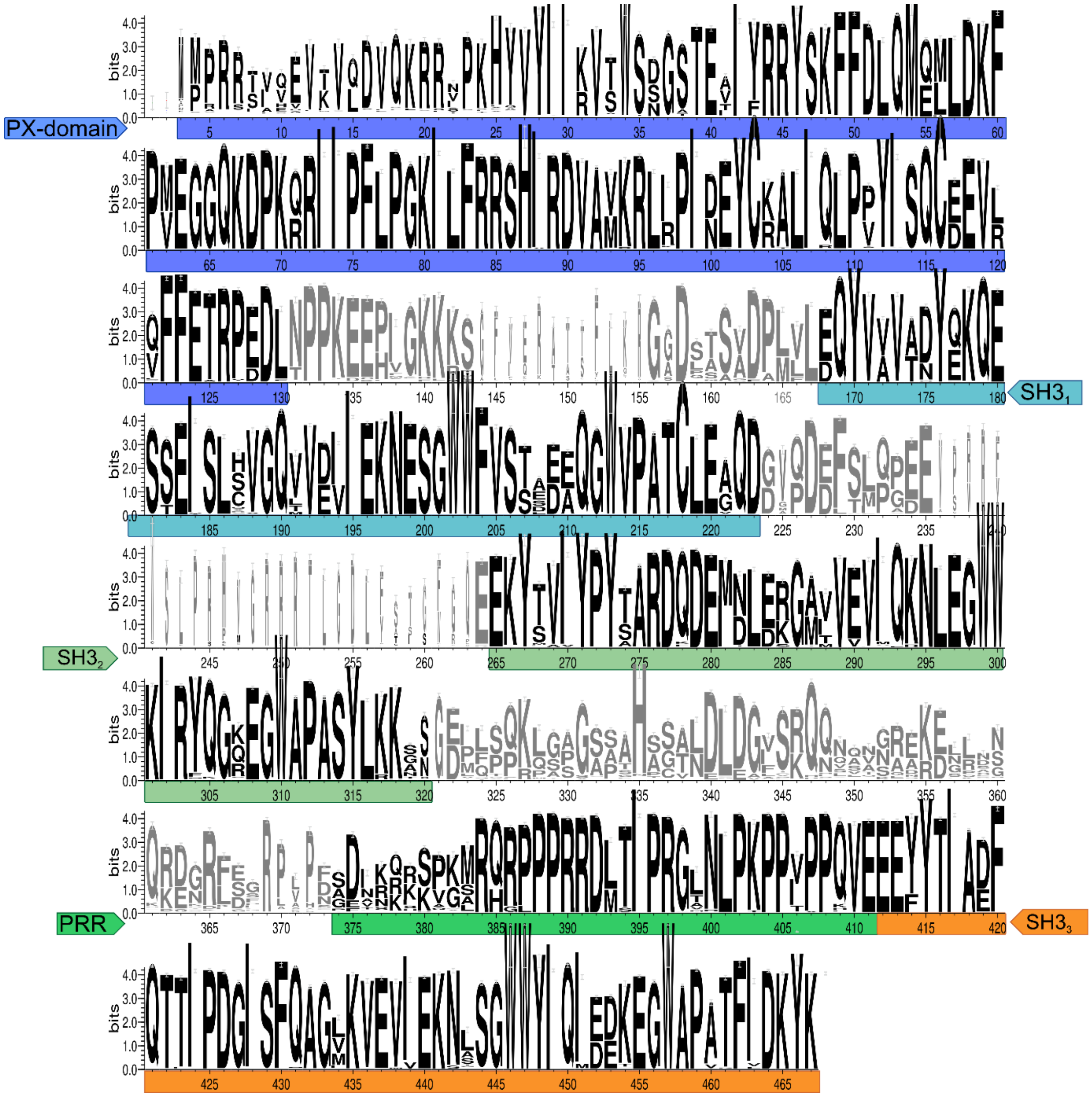

Figure S1. Alignment of the N-terminal region of Tks4 proteins represented by a sequence LOGO. Searching the UniRef100 database [1] for homologous sequences of Tks4 was performed by BLAST[2]. The UniProtKB Entry A1X283 (full length human Tks4) was used as a query sequence. An expectation value (E) threshold of 0.0001 was applied. Gaps were allowed, and the BLOSUM-80 matrix was used for alignment. Unidentified proteins and non-relevant isoforms, such as Tks5, were manually removed from the hits. The remaining 633 hits were aligned by MUSCLE [3]. Sequence logo was generated by WebLogo3 [4].

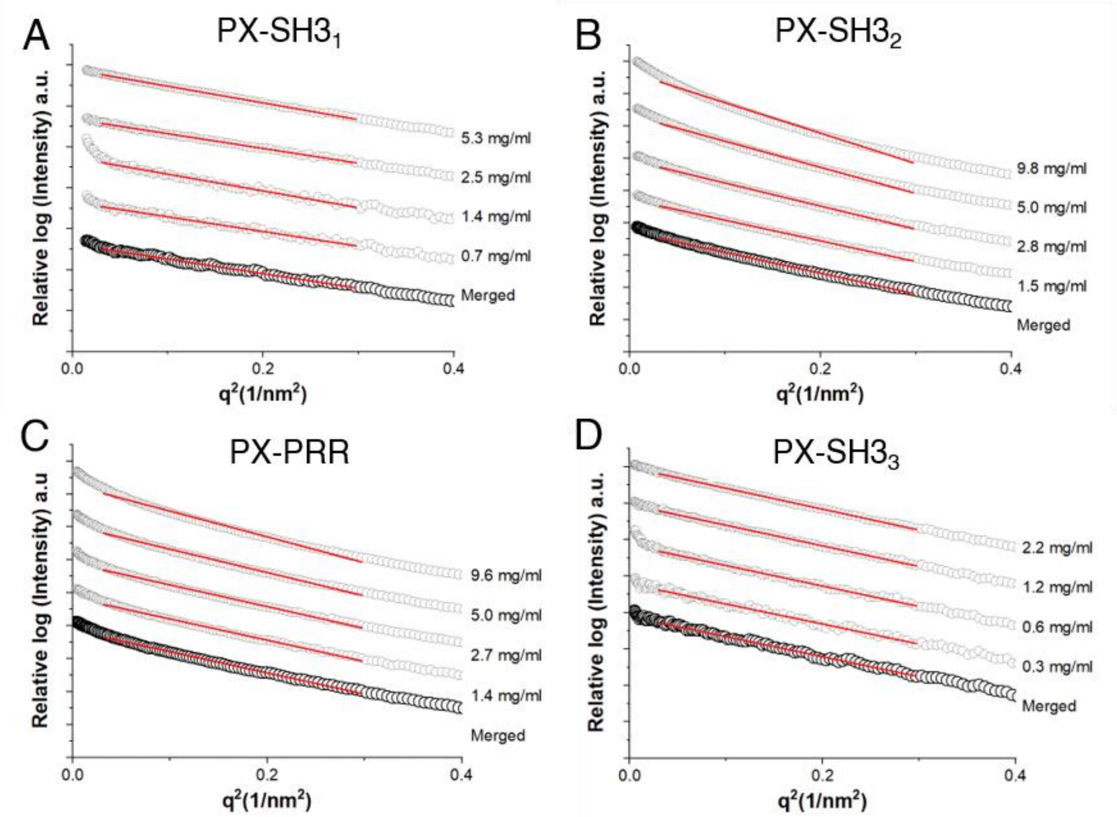

Figure S2. Guinier plot analysis. Stock solutions of the Tks4 fragments PX-$SH3_1$ (A), PX-$SH3_2$ (B), PX-PRR (C), and PX-$SH3_3$ (D) were diluted to different concentrations (with the buffer used for dialysis) as shown. Data in the Guinier region were analyzed by simple linear regression (red lines). Measured data on the entire dilution series were manually merged. The merged curve used for the final R_g calculation is shown in black. Samples showed mostly mild concentration dependent aggregation (moderate in the case of PX-$SH3_2$). The aggregation was reduced at lower concentrations.

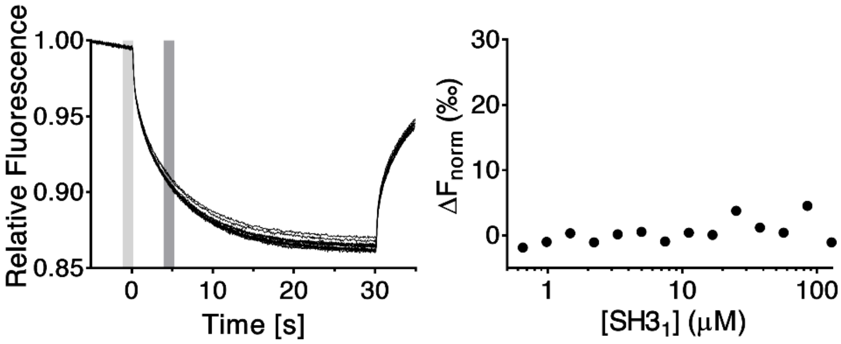

Figure S3. The Tks4 $SH3_3$ and $SH3_1$ domains showed no interaction *in vitro*. Titration of fluorescently labeled Tks4 $SH3_3$ with $SH3_1$ was followed by MST. Left panel: MST traces, right panel: dose-response curve. The change in the normalized fluorescence (ΔF_{norm}) was calculated as the ratio of the average fluorescence in the "cold" (light gray area) and "hot" states (dark gray area) of the system. The experiment was performed as follows: First, the $SH3_3$ fragment was labeled with Nanotemper NT-647 dye. A sixteen-step serial 2:3 dilution of $SH3_1$ was prepared and mixed with a

solution of $SH3_3$, thereby yielding a constant final $SH3_3$ concentration of 128.5 μM. The final $SH3_1$ concentration was varied between 128 μM and 0.3 μM. See the Materials and Methods for further details.

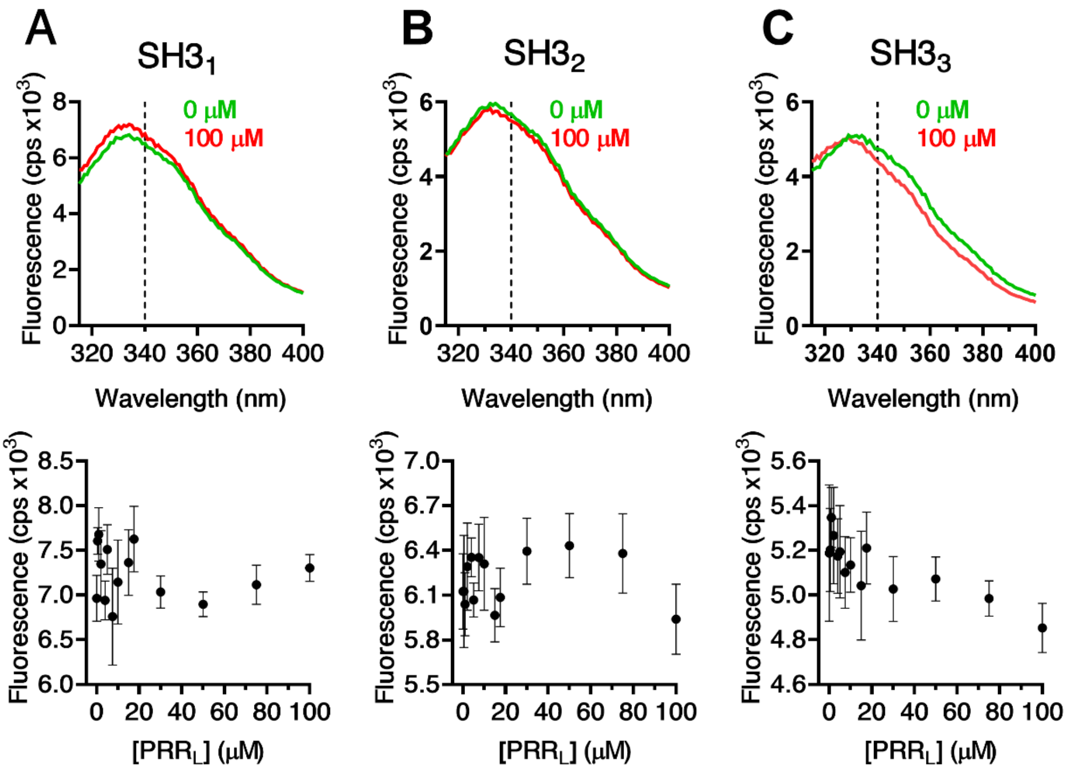

Figure S4. The PRR_L fragment of Tks4 showed no detectable interaction with $SH3_1$ (A), $SH3_2$ (B), or $SH3_3$ (C). Top panels: intrinsic Trp-fluorescence emission spectra of the SH3 domains in the presence (red) and absence (green) of PRR_L (at 100 μM). Vertical dashed lines represent the wavelength (340 nm) where complex formation was followed in other tryptophan fluorescence-based titration experiments (see Figure 3A). Bottom panels: dose-response curves (fluorescence at 340 nm). Error bars depict the standard deviation of three independent measurements. Titration experiments were performed as follows: First, a sixteen-step serial 2:3 dilution of PRR_L was prepared in PBS containing 0.5 mM TCEP and 0.05% (w/v) TWEEN20. Next, stock solutions of $SH3_1$, $SH3_2$, and $SH3_3$ were added to the diluted PRR_L solutions yielding a final (constant) SH3 concentration of 3 μM in all experiments. The final concentration of PRR_L varied between 0.5 μM and 100 μM. Titrations were followed at 340 nm by using a BioTek Cytation 3 instrument with an excitation wavelength of 298 nm.

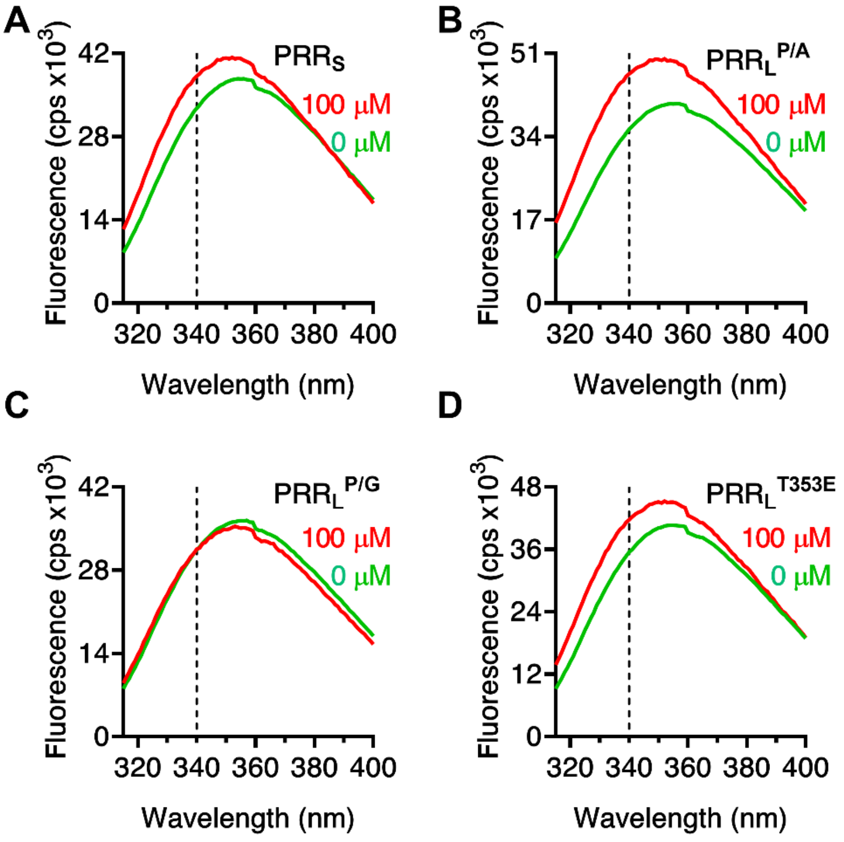

Figure S5. Intrinsic tryptophan fluorescence emission spectra of Tks4 $SH3_1$-$SH3_2$ in the pres-ence (c = 100 μM; red) and absence (green) of PRR_S (A), $PRR_L^{P/A}$ (B), $PRR_L^{P/G}$ (C), and PRR_L^{T353E} (D). Vertical dashed lines represent the wavelength (340 nm) where complex formation was followed in titration experiments (see Figure 4).

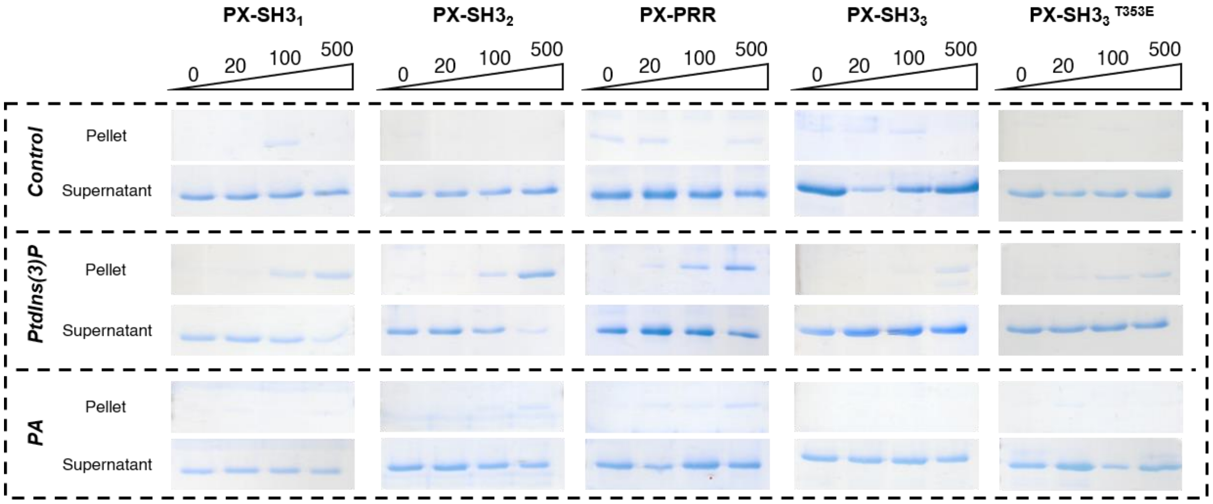

Figure S6. Representative images used for gel densitometry in liposome co-sedimentation assays. Numbers represent the total lipid concentration in μM. PtdIns(3)P and PA were present in liposomes at 5% (mol/mol).

References

1. Suzek, B.E.; Wang, Y.; Huang, H.; McGarvey, P.B.; Wu, C.H. UniRef clusters: A comprehensive and scalable alternative for improving sequence similarity searches. *Bioinformatics* **2015**, *31*, 926–932, doi:10.1093/bioinformatics/btu739.
2. McGinnis, S.; Madden, T.L. BLAST: At the core of a powerful and diverse set of sequence analysis tools. *Nucleic Acids Res.* **2004**, *32*, W20–W25, doi:10.1093/nar/gkh435.
3. Edgar, R.C. MUSCLE: Multiple sequence alignment with high accuracy and high throughput. *Nucleic Acids Res.* **2004**, *32*, 1792–1797, doi:10.1093/nar/gkh340.
4. Crooks, G.E.; Hon, G.; Chandonia, J.-M.; Brenner, S.E. WebLogo: A sequence logo generator. *Genome Res.* **2004**, *14*, 1188–1190, doi:10.1101/gr.849004.
